# Perpendicular reading of single confined magnetic skyrmions

**DOI:** 10.1038/ncomms9541

**Published:** 2015-10-16

**Authors:** Dax M. Crum, Mohammed Bouhassoune, Juba Bouaziz, Benedikt Schweflinghaus, Stefan Blügel, Samir Lounis

**Affiliations:** 1 Microelectronics Research Center, The University of Texas at Austin, 10100 Burnet Road, Austin, Texas 78758, USA; 2 Peter Grünberg Institut and Institute for Advanced Simulation, Forschungszentrum Jülich and JARA, D-52425 Jülich, Germany

## Abstract

Thin-film sub-5 nm magnetic skyrmions constitute an ultimate scaling alternative for future digital data storage. Skyrmions are robust noncollinear spin textures that can be moved and manipulated by small electrical currents. Here we show here a technique to detect isolated nanoskyrmions with a current perpendicular-to-plane geometry, which has immediate implications for device concepts. We explore the physics behind such a mechanism by studying the atomistic electronic structure of the magnetic quasiparticles. We investigate from first principles how the isolated skyrmion local-density-of-states which tunnels into the vacuum, when compared with the ferromagnetic background, is modified by the site-dependent spin mixing of electronic states with different relative canting angles. Local transport properties are sensitive to this effect, as we report an atomistic conductance anisotropy of up to ∼20% for magnetic skyrmions in Pd/Fe/Ir(111) thin films. In single skyrmions, engineering this spin-mixing magnetoresistance could possibly be incorporated in future magnetic storage technologies.

## Abstract (web summary)

Magnetic skyrmions are particle-like configurations which can emerge in the magnetization of materials possessing a chiral exchange interaction. Here, the authors demonstrate how spin-mixing magnetoresistance can allow for the reliable perpendicular detection of single nanoscale skyrmions in Pd/Fe/Ir(111) thin films.

## Graphical abstract

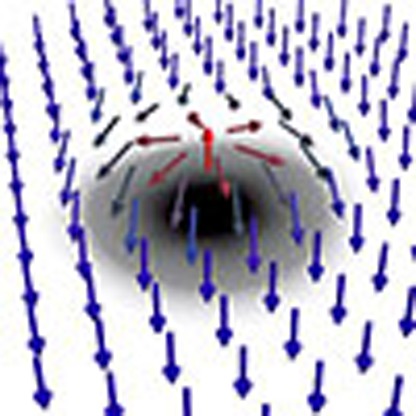

Silicon complimentary metal-oxide–semiconductor^1^ compatible magnetic devices represent the current state-of-the-art in information data storage circuits^2^. In such devices, the information is encoded by manipulation of different spatial magnetic domains, and the data are read by sensing the variation in the magnetoresistance as a function of the magnetization direction^3^. Typically, the different spatial magnetic domains are homogeneous ferromagnetic domains separated by domain walls, but more exotic magnetic configurations, such as magnetic skyrmions, may be ultimately preferred in device applications.

Magnetic skyrmions, for topological reasons^4^, are relatively robust particle-like field configurations^5^,^6^, stable even up to near-room temperature^7^, and could possibly be information carriers or memory storage units in future information technology nodes. Incorporation of magnetic skyrmions instead of domain walls may improve device performance and scaling possibilities^8^. Domain walls are sensitive to defect pinning^9^,^10^,^11^ while skyrmions typically are not^12^,^13^,^14^. This explains reports of skyrmions being moved by electric current densities orders of magnitude smaller than domain walls^15^,^16^ while achieving smaller yet sizeable magnitudes of velocity^14^,^17^,^18^,^19^. Smaller current densities mean that skyrmion-based devices would dissipate less power than domain wall-based devices, and possibly meet stringent power requirements for future deeply scaled technologies^20^.

Recent work has shown more subtle defect-pinning dynamics in skyrmions, however. In certain spin-polarized electric current regimes, skyrmions may be trapped by point magnetic defects^21^, and drift mobilities of skyrmions in some realistic films more closely resemble those of domain walls^22^.

Regardless of defect pinning (and therefore power considerations), moving from domain walls to skyrmions is attractive from a dimensional scaling point of view, as single skyrmions can be confined at will, and their shape and size controlled with an external magnetic field down to diameters <5 nm (ref. 23).

With this in mind, an interesting device scaling route is an extension^8^ of the racetrack memory^24^ to incorporate single^25^,^26^ confined magnetic skyrmions instead of domain walls. Such a concept constitutes an ultimate scaling alternative in terms of packing density, speed and power consumption^8^. Consider a metallic thin-film magnetic heterostructure^26^,^27^ where sub-5 nm chiral skyrmionic quasiparticles are generated (via materials engineering and external magnetic fields) and moved laterally along a magnetic racetrack by in-plane currents, which has been shown experimentally^15^,^16^,^28^. The bit-wise data would be encoded by out-of-plane currents which create or annihilate the individual skyrmions, thus setting or resetting the bit-state. This has also been shown experimentally^26^; thus, two important ingredients to a viable skyrmion racetrack memory system, lateral bit-wise movement and set/reset of each bit-state, have been established experimentally.

But how can one read each bit-state? Current-in-plane detection of skyrmions has been shown experimentally^28^,^29^,^30^ and understood theoretically as a topological Hall effect^31^,^32^, but may be costly in terms of power consumption and difficult to fabricate in terms of device geometries. A better option would be the direct detection of the nanoskyrmions via current perpendicular-to-plane (CPP)^33^,^34^ geometries.

Near a skyrmion, the microscopic magnetoresistance varies as a function of the atomic magnetization direction due to the spin mixing of electronic states on neighbouring sites. Considering a skyrmion's central spin-flipped atom, its local magnetic environment is inequivalent from those atoms in the ferromagnetic background. Thus, in the presence of noncollinearity, one could expect already a conductance anisotropy in the centre of the skyrmion due to the change in the electronic structure relative to the ferromagnetic (FM) state. In addition, the heavy metal substrate induces a large spin–orbit interaction (SOI), coupling the local magnetization to the real-space direction, further modifying the electronic structure as a function of magnetization rotation relative to the substrate plane. This tunnelling anisotropic magnetoresistance (TAMR)^35^ effect has been studied in detail but typically for homogeneous magnetic domains (either different ferromagnetic domains^35^,^36^ or homogeneous spin spirals^37^), not inhomogeneous spin textures such as skyrmions.

In this Article, we demonstrate a spin-averaged electrical detection mechanism for single skyrmions in a CPP-geometry. This all-electrical signature is a departure from typical experiments which rely on spin-polarized injection to detect magnetic structures, and thus inherently an improvement from a device application perspective. Our mechanism is viable due to the reliable modification of the local tunnelling magnetoresistance into the sample near the vicinity of a skyrmion. Using single confined magnetic skyrmions as examples, we explain this combined (noncollinear and SOI induced) spin-mixing magnetoresistance in terms of fully self-consistent calculations of entire skyrmions altogether from first principles, rather than employing extrapolated models as commonly done^27^,^38^,^39^,^40^. Here we have direct access to the electronic structure of not just each skyrmion as a whole, but even of the states decaying into the vacuum, which are essential to the tunnelling conductance. We find a rather large atomistic conductance anisotropy of up to ∼20% (∼10%) for magnetic skyrmions in Pd/Fe/Ir (Pd/Pd/Fe/Ir) magnetic thin films, which potentially could be detected in a realistic device exploiting a CPP-geometry. Developing the physics of this generalized tunnelling spin-mixing magnetoresistance (TXMR) could possibly inspire the design of future nanomagnetic devices based on such a mechanism.

## Results

### System and procedure

**Figure 1:**
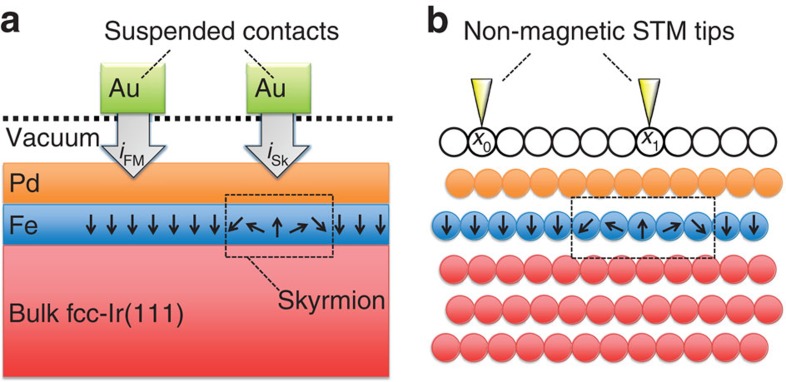
Out-of-plane detection of nanoskyrmions. (**a**) Illustrative heterostructure cross-section for the perpendicular reading of single nanoskyrmions. Due to energy-dependent spin-mixing perturbations to the atomistic electronic structure as a function of position within skyrmions, the electric current relation *i*_FM_≠*i*_Sk_ holds, and therefore the magnetic data information can be sensed in a CPP-geometry. (**b**) Illustrative STM-spectroscopy experiment of fcc-Pd/Fe overlayer on single-crystal fcc-Ir(111) bulk substrate. The tunneling conductance is modified by the combined effects of local magnetic noncollinearity and substrate-induced spin–orbit interaction. For similar physical reasons as **a**, the tunnelling conductance at position *x*_0_ is different from that at position *x*_1_.

Thin-film systems containing single nanoscopic skyrmions make a dynamic combination from an engineering perspective, due to the robustness of the skyrmion data carriers and the ultimate scalability of future fabricated devices. Such an auspicious system is schematically illustrated in Fig. 1a. In the upcoming discussions, we will show how the tunnelling current between a suspended metal contact through vacuum depends on the noncollinear magnetic state-of-phase below. Suspended metal contacts are possible with state-of-the-art fabrication techniques^41^, but one could also imagine tunnelling through a weakly interacting two-dimensional insulator, such as hexagonal boron nitride (hBN) or molybdenum disulfide (MoS_2_). Such a process can be intimately understood in a nonspin-polarized scanning tunnelling microscopy (STM) experiment (Fig. 1b).

In this study, we consider two magnetic thin-film heterostructures similar to Fig. 1b purely from *ab initio*: fcc overlayers of Pd/Fe and Pd/Pd/Fe on single crystal bulk fcc-Ir(111). These systems are attractive for a number of reasons. First, they generate large Dzyaloshinskii–Moriya interactions (DMIs)^42^,^43^,^44^, whose competition with the isotropic exchange interaction *J* determines the size and chirality of the skyrmions^8^. DMIs are large here because of the strength and nature of the inversion symmetry breaking in the heterostructures. At the Fe/Ir(111) interface, a large SOI in the underlying heavy metal substrate, here Ir(111), is relatively uncompensated by the overlayer Pd/Fe or Pd/Pd/Fe interface, leading to a large DMI vector preferentially in the plane of Fe, denoted by **D**. The ratio of |**D**|/*J*, along with an external magnetic field, can stabilize isolated skyrmions with diameter *D*_Sk_≈1–5 nm in size, and has been shown experimentally^23^,^26^. Second, by choosing a double-Pd overlayer (Pd/Pd/Fe/Ir) versus a single-Pd overlayer (Pd/Fe/Ir), one can alter the exchange interactions in Fe due to the modified nature of the interface hybridization and electronic charge transfer (Supplementary Note 1 and Supplementary Tables 1 and 2). We investigate this effect to illuminate conceptual studies where other overlayer combinations and materials are used to engineer the size, shape and stability of the isolated skyrmions^27^,^39^,^40^.

We focus specifically on single skyrmions and do not investigate networks or lattices of skyrmions. We perform self-consistent density functional theory (DFT) calculations based on a full-potential Green function formalism including SOI^45^, which allows a perfect embedding of real-space defects, such as isolated skyrmions, into the ferromagnetic background system. Additional specifics of our computational scheme are given in the Methods section.

### Noncollinear inhomogeneity in nanoskyrmions

**Figure 2:**
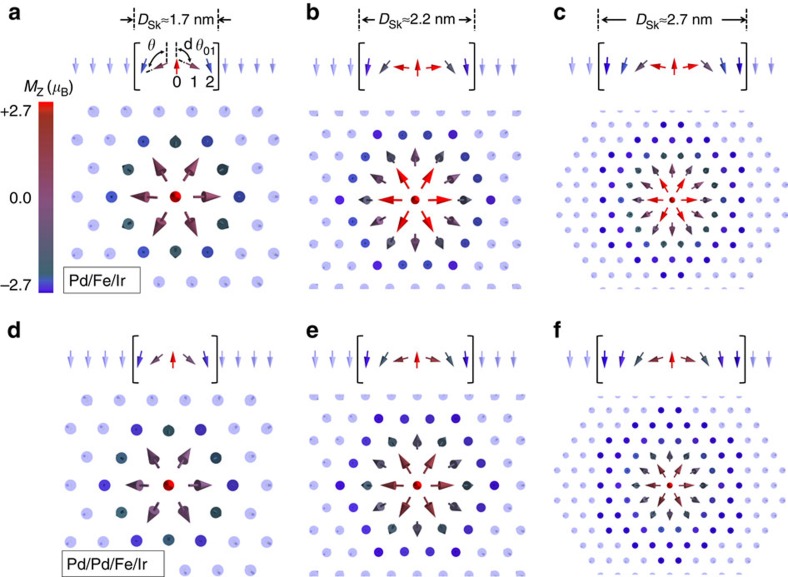
Real-space relaxation of nanoskyrmions with increasing size. (**a**–**c**) Plots of axisymmetric cycloidal spin whirls inside a magnetically active Fe-layer centred about increasingly larger skyrmionic defects in fcc-Pd/Fe overlayer on fcc-Ir(111) bulk substrate. Confining spins in the FM background are shown transparent. We define *θ* as the typical polar angle with the vertical and d*θ* as the difference in polar angle between adjacent pairwise atoms. (**d**–**f**) Again but in fcc-Pd/Pd/Fe overlayer on fcc-Ir(111) bulk substrate. The colour bar in **a** represents the magnitude of the *z*-component of the magnetization for each spin in **a**–**f**.

Before coming to the essential physics of the TXMR effect, we first self-consistently relax different sized nanoskyrmions in otherwise ferromagnetic backgrounds (Fig. 2), in both single- and double-Pd overlayer material stacks. We control the size of the skyrmionic defects by allowing different finite numbers of atoms to relax their magnetic moments in size and direction after the central atom has been spin flipped as an initial condition. We investigate three different realistic skyrmion sizes: *D*_Sk_≈1.7, 2.2, and 2.7 nm in diameter. The spin textures exhibit a fixed and unique rotational sense as demanded by the DMI, which seeks energy gain by torquing the moments to rotate with respect to their neighbours. These structures are cycloidal and radial in nature as expected for magnetic thin films. Thus our theoretical calculations are consistent in generating realistic nanoskyrmions which have been experimentally detected using magnetic spin-polarized currents^23^,^26^.

We illustrate the spin-moment global rotation versus the vertical (polar angle *θ*) of each atom and the pairwise difference between adjacent polar angles (d*θ*). We will show that the spin-mixing perturbations to the local-density-of-states (LDOS) are a function of these angular parameters because the relative canting between different pairwise atomic sites varies as a function of space, in addition to the absolute canting relative to the substrate. While traversing across the diameter of any of the nanoscopic skyrmions shown in Fig. 2, we mention that d*θ* itself is not constant between different nearest neighbour atom pairs, such that there exists an inhomogeneity on the atomic scale in the rotation of the magnetization direction with respect to the substrate plane. Furthermore, these inhomogeneities themselves are a function of diameter when comparing skyrmions of different sizes (Fig. 2a–c).

### Electronic structure of isolated confined skyrmions

We now move to establish the physics behind the TXMR effect within a scanning tunnelling microscopy/spectroscopy (STM or STS) experiment employing a nonspin-polarized tip, for which according to the Tersoff–Hamann model^46^, the differential conductance d*I*/d*V* is proportional to the LDOS of the sample, calculated at the tip position, **R**_tip_, and the given bias energy *E*_bias_:


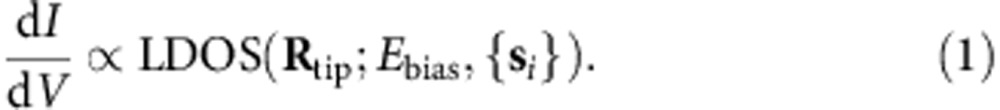


The LDOS depends on the configuration {**s**_*i*_} of atomic spins of the sample relative to each other, for example, in terms of d*θ*_*ij*_=*θ*_*j*_−*θ*_*i*_ for all atom pairs (*i*,*j*), and relative to the lattice in terms of the absolute polar angle *θ*_*i*_. The transport phenomenon related to the latter is known as the TAMR^35^, an effect related to spin mixing due to the SOI. The former results from the spin-mixing hybridization of majority and minority states due to noncollinearity. Both can be subsumed as TXMR. Common to both is that the probability of tunnelling into majority and minority states depends on angles. The difference is that the impact on the electronic structure due to noncollinearity can be of first order and thus larger than that due to the SOI, which is typically of second order. While the SOI is nominally of the order of tens of meV, noncollinear interactions are mediated by the exchange splitting of the electronic states (∼few eVs) and the strength of their hybridization, which here is of the order of hundreds of meV (see Supplementary Notes 2 and 3 and Supplementary Figs 1 and 2 for more details). Also the appearance of both are different. For example, the TXMR due to noncollinearity in a homogeneous magnetic spiral is the same across the spiral, because d*θ*_*ij*_=d*θ* for all atom pairs (*i*,*j*), but different for spirals of different pitches under the transformation d*θ*→d*θ*′. In contrast, the TAMR is modulated across the spiral^37^ as *θ*_*i*_ changes from atom-to-atom. Thus, the TXMR is used to measure conductance differences between two different magnetic states such as the difference between a skyrmion and the FM-state, but can also be used to resolve magnetization inhomogenieties inside complex spin textures such as skyrmions or domain walls.

**Figure 3:**
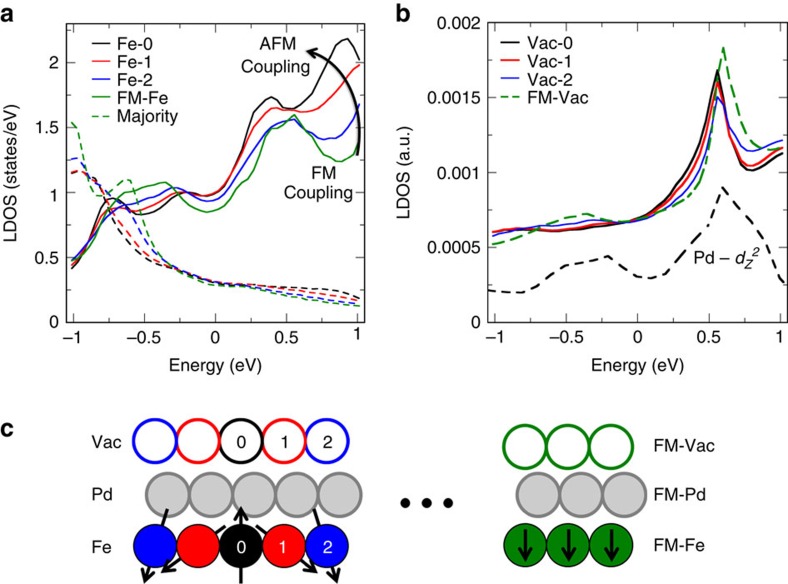
Electronic structure of a realistic *D*_Sk_≈1.7 nm skyrmion in Pd/Fe/Ir. (**a**) LDOS in the magnetically active Fe-layer resolved into minority (solid) and majority (dashed) spin channels. The resonance peak near *E*≈0.5 eV in the FM background (green) shifts in energy when approaching the centre of the skyrmion (black). (**b**) The modification of the electronic structure in Fe contributes to a strong resonance in the LDOS in vacuum via hybridization through surface Pd states. Arbitrary units are used so as to include in the same plot the nature of the 
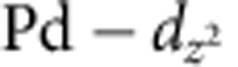
 surface-state (black dashed), whose resonance peak near 0.5 eV survives in the vacuum. (**c**) Illustrative legend for **a**,**b** where the numbered spheres represent a line extending radially from the skyrmion's centre. The vacuum domains are represented by empty spheres. FM-Fe, FM-Pd and FM-Vac represent the unperturbed background ferromagnet.

Since the spin-mixing perturbations due to noncollinearity and SOI are magnetic in nature, we show in Fig. 3a the spin-dependent LDOS in the magnetically active Fe layer as a function of the atomic position for the *D*_Sk_≈1.7 nm skyrmion. For brevity we plot only the Pd/Fe/Ir case (see Supplementary Note 4 and Supplementary Fig. 3 for the larger *D*_Sk_≈2.2 nm case and Supplementary Note 5 and Supplementary Figs 4 and 5 for the double-Pd cases). We note that the majority and minority spin channels are given in the local spin frame of reference for each Fe atom. The colour coding of the plot, which corresponds to different atoms extending radially from the skyrmion's centre, is explained in Fig. 3c. The energy zero is the Fermi energy, *E*_F_=0.

The resonant states between 0.5 and 1.0 eV above *E*_F_ are of Fe *d*-band minority spin character and consist of 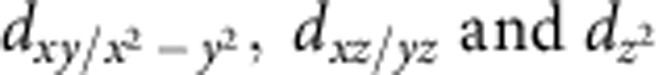 states. These states hybridize with the *sp* states in the Pd overlayer and give rise to 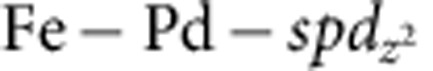 hybrid states, named in short 
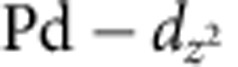
 states localized in the Pd overlayer film around *E*≈0.5 eV, as shown in Fig. 3b (black-dashed curve). It is clear that the surface layer 
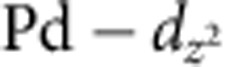
 state (shown only for the background FM-Pd-surface film), which has the proper orbital symmetry to decay slowly transverse to the substrate, controls the electronic structure in vacuum as a function of energy, characterized by a strong resonance in the vacuum LDOS. An all-electrical STS measurement will be sensitive to this vacuum resonance peak.

The origin of this resonance and its behaviour on rotation can be understood by analysing the energy window near *E*≈0.5 eV in Fig. 3a, where one can see the resonance peaks shifting in energy in the Fe skyrmion LDOS as a function of position. The green curve, which represents the ferromagnetic state of the background Fe film, shows an electronic structure consistent with Fe minority *d*–*d* hybridization when adjacent atoms couple ferromagnetically (Supplementary Note 4). Conversely, moving towards the centre of the skyrmion, the quantization axes between two neighbouring atoms become different, and majority states of one atom can hybridize with minority states of the second. This effect is especially pronounced at the central spin-flipped atom (black curve), where the resonance peak has shifted lower in energy—as expected for antiferromagnetic coupling (Supplementary Note 4). We reproduce these effects within the context of a simple model, where we can qualitatively predict the change in LDOS as a function of the noncollinear magnetization rotation parameter d*θ* as defined in Fig. 2a (Supplementary Notes 2 and 3).

The energy-dependent disturbance to the LDOS resonance peaks as a function of position moving radially along the skyrmion will manifest as a perturbation to the local electrical conductivity, and is the physical basis for the space-dependent TXMR effect.

### All-electrical skyrmion detection

We now define the TXMR and make predictions for future experimental observation of the effect. The TXMR is the percent deviation of the local conductance from a reference conductance due to the spin mixing from noncollinearity and SOI. As long as the magnetic state under consideration has a different noncollinearity than the reference state, there will be a TXMR. If one is interested in the spatial resolution of a complex spin texture (ignoring SOI), however, then an additional inhomogeneity within the noncollinearity is required, as is the case for nanoskyrmions.

The TXMR is by definition measured in vacuum. Here, we choose the reference to be somewhere far from the skyrmion in the FM background. Then the normalized TXMR measured at site *r* is


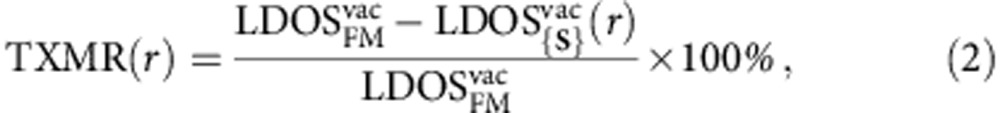


where 
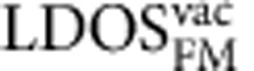
 is the LDOS in the vacuum just above the FM, and 
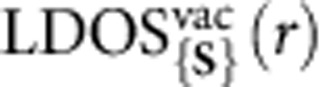
 is the LDOS of the complex spin texture in the vacuum just above site *r*.

Integrating the TXMR over the entire device injection boundary, over all energies up to the bias energy *eV*_bias_, would give a measure of the total change in conductance, and would be the state-of-bit detection mechanism in a CPP-TXMR device like discussed in Fig. 1a. In an STS experiment, however, the effect could be amplified by selecting specific energy windows where the TXMR were largest as a function of position.

**Figure 4:**
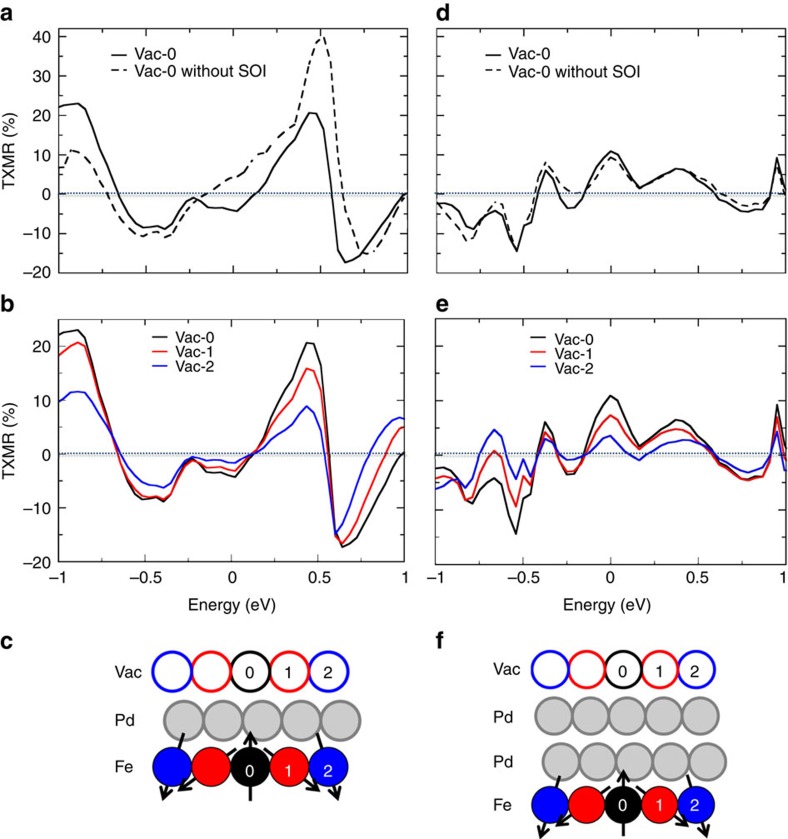
Tunnelling spin-mixing magnetoresistance. (**a**) Energy-resolved TXMR signals measured at the skyrmion's core comparing the effects of SOI in a *D*_Sk_≈1.7 nm skyrmion in Pd/Fe/Ir. (**b**) TXMR signals again, but for a line of atoms extending radially from the centre of the skyrmion, illustrating the spatial dependence of the effect. (**c**) Illustrative legend for **a**,**b** where the spheres are numbered and colour coded to identify the appropriate curves to the corresponding vacuum domains above the skyrmion. (**d**–**f**) Plots corresponding to **a**–**c** but in the Pd/Pd/Fe/Ir system.

In Fig. 4a,d we show the energy-resolved TXMR of the *D*_Sk_≈1.7 nm skyrmion's central spin-flipped vacuum site, with and without SOI, for the single- and double-Pd cases, respectively. We notice a sizeable TXMR effect for both systems. This holds true for all skyrmions that we studied, noting a small size-dependence of the effect which varies weakly as a function of *D*_Sk_ (Supplementary Note 6 and Supplementary Fig. 6).

TXMR signals in the different single-Pd and double-Pd material systems vary from skyrmion-to-skyrmion, however. Since the SOI arises from the Fe–Ir interface, the impact of SOI is much more pronounced in the single-Pd system (Fig. 4a), where the TXMR can peak out at an impressive ∼40% when ignoring spin–orbit coupling, but decreases down to ∼20% when SOI is included. Interestingly, the spin mixing due to SOI is to compete with the effects due to inhomogeneous noncollinearity, reducing the overall TXMR signal. In the double-Pd system, most of the TXMR signal is due to inhomogeneous noncollinearity, with a small contribution coming from the SOI (Fig. 4d).

In Fig. 4b,e we plot the spacial variation of the TXMR signal, which differs significantly from atom-to-atom within the same skyrmion. We see that the TXMR effect is reduced when approaching the edge of the skyrmion (Fig. 4b,e, blue curves), since the effective noncollinearity is reduced as the complex spin texture fades into the ferromagnetic background.

**Figure 5:**
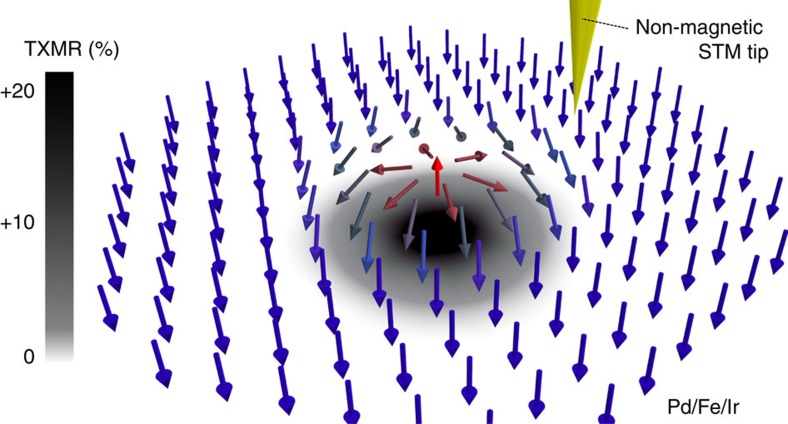
Perpendicular reading of single magnetic skyrmions. Expected STS-signal when approaching a skyrmionic defect in the single-Pd case. The electrical contrast has been projected onto the plane below the skyrmion. Near the injection energy *eV*_bias_≈0.5 eV, there are about ∼20% fewer tunnelling states in the skyrmion's core compared with the unperturbed FM environment. This increases the local magnetoresistance, allowing for the reliable spin-averaged electrical detection of skyrmions in a CPP-geometry.

The vacuum resonance we found in Fig. 3b appears now as a large TXMR signal at the same energy in the Pd/Fe/Ir(111) system (Fig. 4a,b). Thus, an experimentalist probing the surface in a STS experiment, having set the ripple-bias voltage near *V*_bias_≈0.5 V, would see an electrical contrast as visualized in Fig. 5 when approaching a skyrmion of similar size in the single-Pd heterostructure.

Within a reasonable bias voltage range, the TXMR effect is smaller in the double-Pd case (∼10%) when compared with the single-Pd case (∼20%). The additional Pd overlayer changes the resonance nature around 0.5 eV compared with the single-Pd case, and states with a high tunnelling cross-section into the vacuum are distributed over a wider energy (Supplementary Note 5). As a consequence, the TXMR is reduced by nearly half when compared to the single-Pd case, and a bias energy near −0.8 eV is experimentally more favourable.

## Discussion

We have studied realistic and experimentally observable confined nanoskyrmions within metallic thin films of Pd/Fe/Ir(111) and Pd/Pd/Fe/Ir(111) completely from first principles. We established how the combined effects of local inhomogeneous magnetic noncollinearity and SOI in nanoskyrmions can alter the atomistic electronic structure in a magnetically active Fe film, and, via hybridization with additional surface layers, the electrons which tunnel into the vacuum.

The change in the LDOS can be understood in terms of the rotation parameters of the magnetic moment of the considered atom. The largest spin-mixing contribution comes from noncollinearity and depends on the relative canting between magnetic moments on neighbouring sites, d*θ*. The dependence on the absolute polar angle of the magnetic moment with respect to the substrate, *θ*, comes in as a second order term to the change in the LDOS, but can become important if the impact of the SOI is large.

Finally, we have shown in detail how such a physical interplay could induce a sizeable electrical conduction anisotropy as a function of position and energy in realistic nanoskyrmions, up to ∼20% in the single-Pd case. The manifestation of this TXMR effect could possibly be exploited in an all-electrical tunnelling spectroscopy experiment.

In addition, the changes in the magnetoresistance on the nanometre scale of skyrmions can possibly be engineered to design advanced magnetic memory devices. Typical memory circuits require at least one control device (either transistor or diode) in each memory cell. Instead, technologies based on spin mixing in single skyrmions could have potentially hundreds of bits stored in nanometre-sized magnetic racetracks needing only a single read-out element to detect the contents of each array (Supplementary Note 7 and Supplementary Fig. 7).

Such a mature magnetic device technology would have to be relatively impervious to interface quality, film surface roughness, and various point-defect impurities, however. Now while the TXMR effect reported here in this work lead to an impressive magnitude of conduction anisotropy in epitaxial magnetic thin films, future challenges will be related ultimately to the robustness of the TXMR in realistic devices.

## Methods

### Computational details

The electronic structure was determined employing DFT in the local spin density approximation^47^. Calculations were executed by means of the screened Korringa–Kohn–Rostoker full-potential relativistic Green function method^45^. A full-potential method is important to accurately describe the nature of the complex spin texture and rapidly decaying vacuum states of the tunnelling electrons.

For the calculations we chose an angular momentum cutoff of *l*_*max*_=3 for the orbital expansions of the Green functions. The energy contour for numeric integration of the spin and charge density contained 40 grid points in the upper complex plane (including seven Matsubara poles) with a Brillouin zone mesh of 30 × 30 *k*-points. The FM-slab LDOS and skyrmion impurity cluster LDOS were obtained by one-shot calculations using the FM-state or skyrmion-state as starting points, respectively. We found that increasing the *k*-mesh to 200 × 200 was sufficiently adequate to numerically stabilize the relevant observables.

### Thin-film slab configurations

The magnetic thin-film slab configurations follow, where positive percentages refer to inward relaxation with respect to the Ir(111) interlayer separation. We consider only fcc-stacking in all cases, which is in fact energetically favourable compared with hcp crystal growth^39^.

Pd/Fe/Ir: 44 total layers (3 vacuum+1 vacuum (−1%)+1 Pd (8%)+1 Fe (7%)+1 Ir (1%)+33 Ir+4 vacuum).

Pd/Pd/Fe/Ir: 44 total layers (3 vacuum+1 Pd (−1%)+1 Pd (8%)+1 Fe (7%)+1 Ir (1%)+33 Ir+4 vacuum).

We choose 34 Ir layers since it was the minimum thickness by which we completely decoupled any wave function penetration from top-to-bottom surface. We obtained the relaxation parameters as optimized and reported by Dupé *et al.*^39^

### Calculating whole skyrmions within DFT

To stabilize skyrmions after determining the two-dimensional-FM slabs, the slab Green functions were harvested and a single spin-flipped Fe atom was embedded in the FM background. We then allowed three-layer cylindrical ring-like stacks of atoms within the skyrmion impurity cluster to update their potentials and magnetic moments (Fe-layer+1 Pd-layer above and 1 Ir-layer below). The effect of the FM background was included self-consistently by the slab Green function (*G*_0_), which connects the skyrmion impurity cluster (*G*_imp_) to the host via the Dyson-like equation: *G*_imp_=*G*_0_+*G*_0_Δ*VG*_imp_, where Δ*V* represents the modified atomic potential as compared to the unperturbed slab Green function potential, *V*. In such a manner a real-space defect can be perfectly embedded in an otherwise periodic crystal. After converging the different sized skyrmionic profiles, observables were calculated as mentioned previously.

## Additional information

**How to cite this article:** Crum, D. M. *et al.* Perpendicular reading of single confined magnetic skyrmions. *Nat. Commun.* 6:8541 doi: 10.1038/ncomms9541 (2015).

## Supplementary Material

Supplementary Information Supplementary Figures 1-7, Supplementary Tables 1-2, Supplementary Notes 1-7 and Supplementary References.
