# Supplementary material for: Perpendicular reading of single confined magnetic skyrmions
Source: Nat Commun. 2015 Oct 16;6:8541. doi: 10.1038/ncomms9541 (PMC4634132; doi:10.1038/ncomms9541)
Supplement: Supplementary Information — Supplementary Figures 1-7, Supplementary Tables 1-2, Supplementary Notes 1-7 and Supplementary References. [file ncomms9541-s1.pdf]

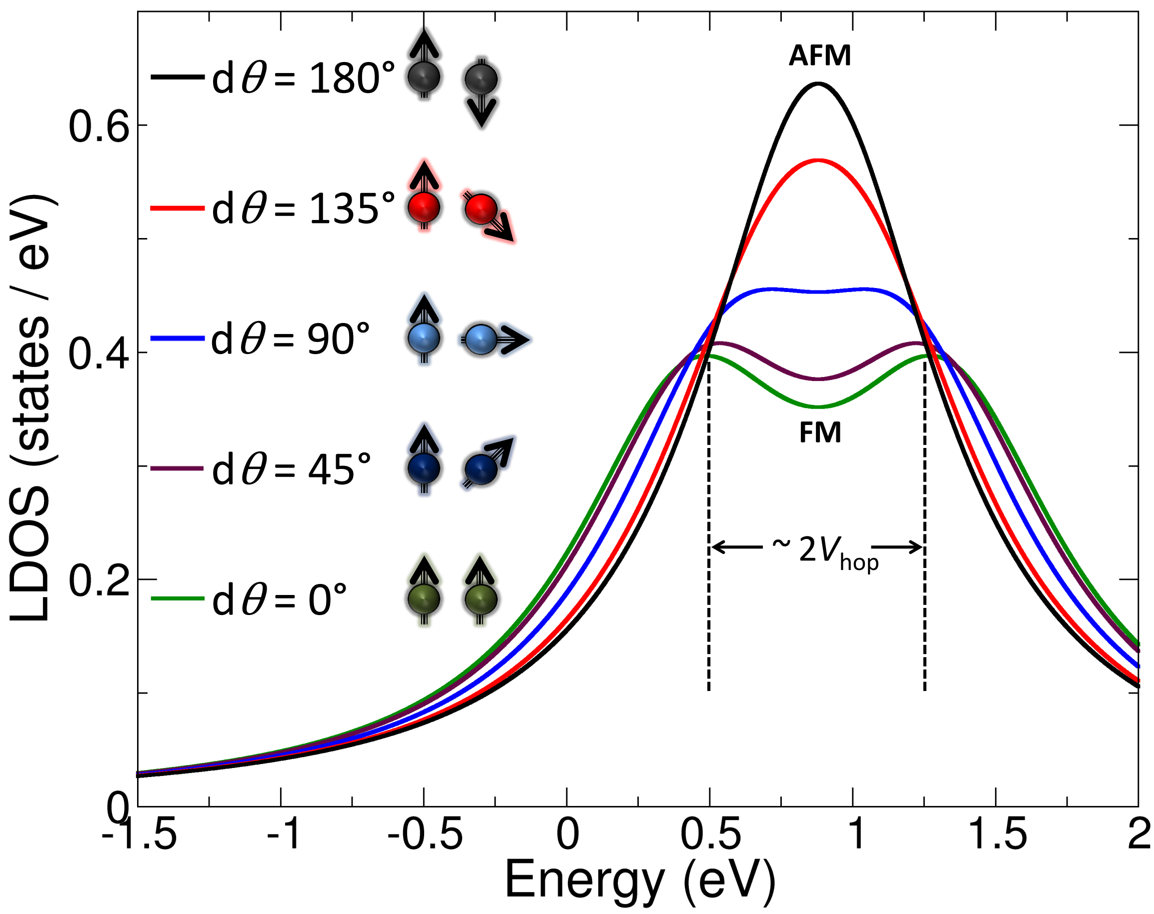

Supplementary Figure 1 | Alexander-Anderson prediction for d-d hybridization. Beginning from dθ=0°, ferromagnetic coupling imposes a hybridization between the two d_{z^2} orbitals localized at the same energy but on neighboring atoms. This produces a splitting into a bonding- and antibonding-state (green curve). Upon rotation, the atoms eventually become antiferromagnetically coupled (black curve). The change in the LDOS can be qualitatively estimated with a $\cos(\mathrm{d}\theta)$ fitting parameter.

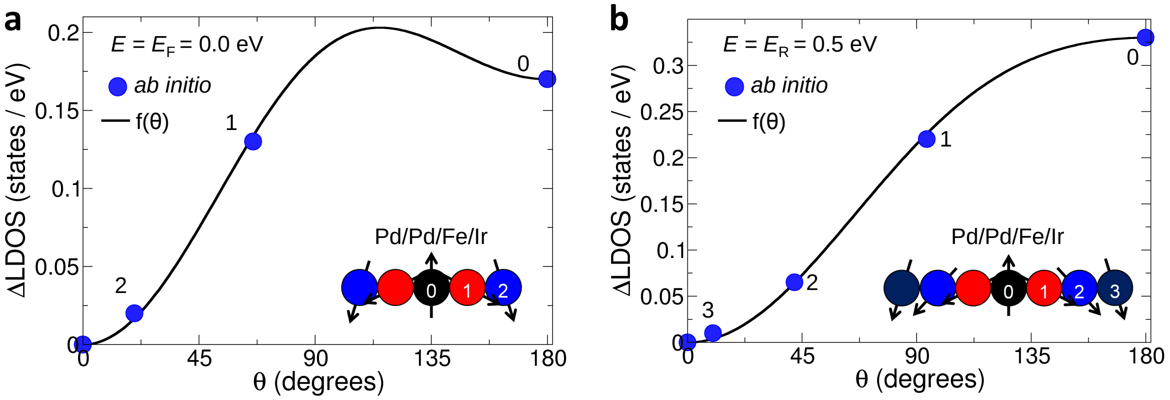

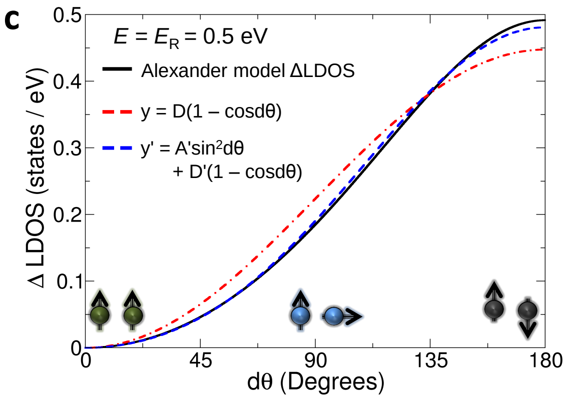

d

	Fe-0	Fe-1	Fe-2	Edge FM
**Θ**	180°	66°	20°	0°
**dθ**	114°	80°	33°	10°

(**a**) Pd/Pd/Fe/Ir: D_{Sk} = 1.7 nm

	Fe-0	Fe-1	Fe-2	Fe-3	Edge FM
**Θ**	180°	94°	42°	10°	0°
**dθ**	86°	69°	42°	21°	5°

(**b**) Pd/Pd/Fe/Ir: D_{Sk} = 2.2 nm

Supplementary Figure 2 | Energy- and spatially-dependent ΔLDOS in skyrmions. (a) Fe-layer atom-by-atom *ab initio* results (blue dots) of the total change in LDOS as a function of θ in a 1.7 nm diameter skyrmion in Pd/Pd/Fe/Ir. ΔLDOS is given relative to the background-FM and is measured at the Fermi energy while including SOI. These results are then fitted to $f(\theta)$ (solid curve). The $f(\theta)$ in (**a,b**) refers to Supplementary Equation 16, while the numbers labeling each dot respresent the corresponding atom in the inset extending radially from the skyrmion core. **(b)** Again, Fe-layer first-principles results (blue dots) of the atomistic change in LDOS in Pd/Pd/Fe/Ir and fitted against $f(\theta)$ (solid curve), but for a larger 2.2 nm skyrmion and measured at the resonant energy $E_{\mathrm{R}} = 0.5$ eV. **(c)** We plot the ΔLDOS as a function of $\mathrm{d}\theta$ given by our two-atom Alexander-Anderson model (black curve) to isolate the non-collinear component of the ΔLDOS. While the main contribution comes from $\cos(\mathrm{d}\theta)$ (red-dashed, referring to Supplementary Equation 15), a higher-order term is required to complete the fit (blue-dashed). **(d)** Rotation parameters as found from the first-principles calculations, referring to (**a**) and (**b**).

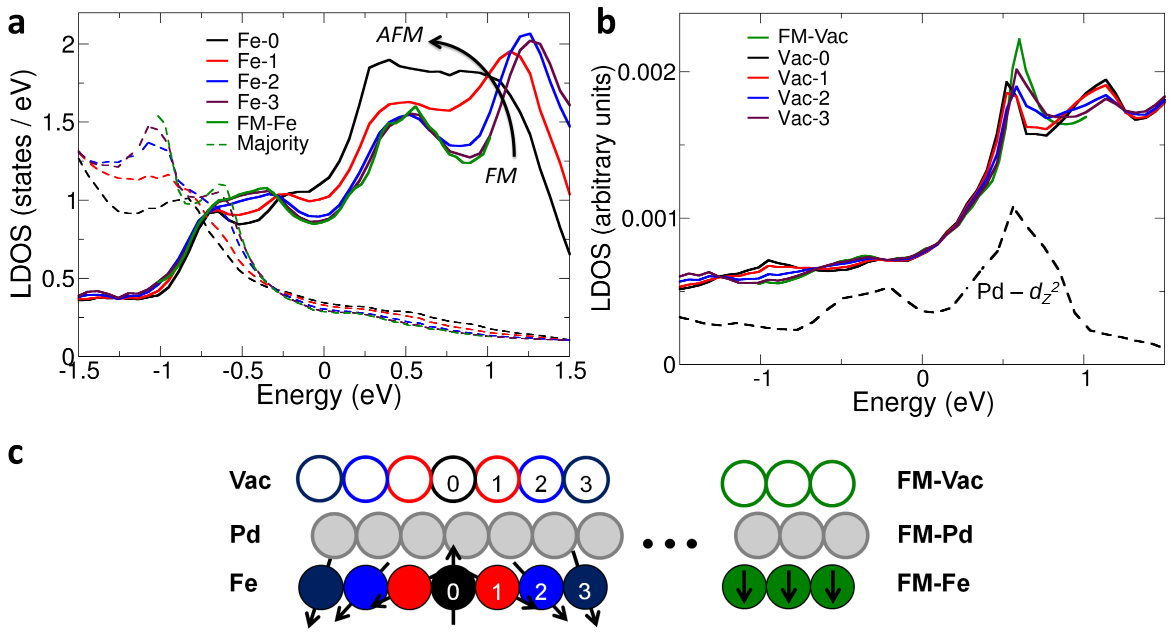

Supplementary Figure 3 | LDOS of a $D_{\mathrm{Sk}} \approx 2.2$ nm skyrmion in Pd/Fe/Ir. (a) Electronic structure in the magnetically-active Fe-layer resolved into minority (solid) and majority (dashed) spin-channels. The splitted-structure of the FM-background (green) is modified due to quasi-AFM interactions when approaching the center of the skyrmion (black). **(b)** The alteration of the LDOS in Fe contributes to a strong resonance in vacuum via hybridization through surface Pd-states. Arbitrary units are used so as to include in the same plot the nature of the Pd-d_{z^2} surface-state (black-dashed), whose resonance-peak near 0.5 eV survives in the vacuum. **(c)** Illustrative legend for (**a**, **b**) where the numbered spheres represent a line extending radially from the skyrmion's center. The vacuum domains are represented by empty spheres. FM-Fe, FM-Pd, and FM-Vac represent the unperturbed background ferromagnet.

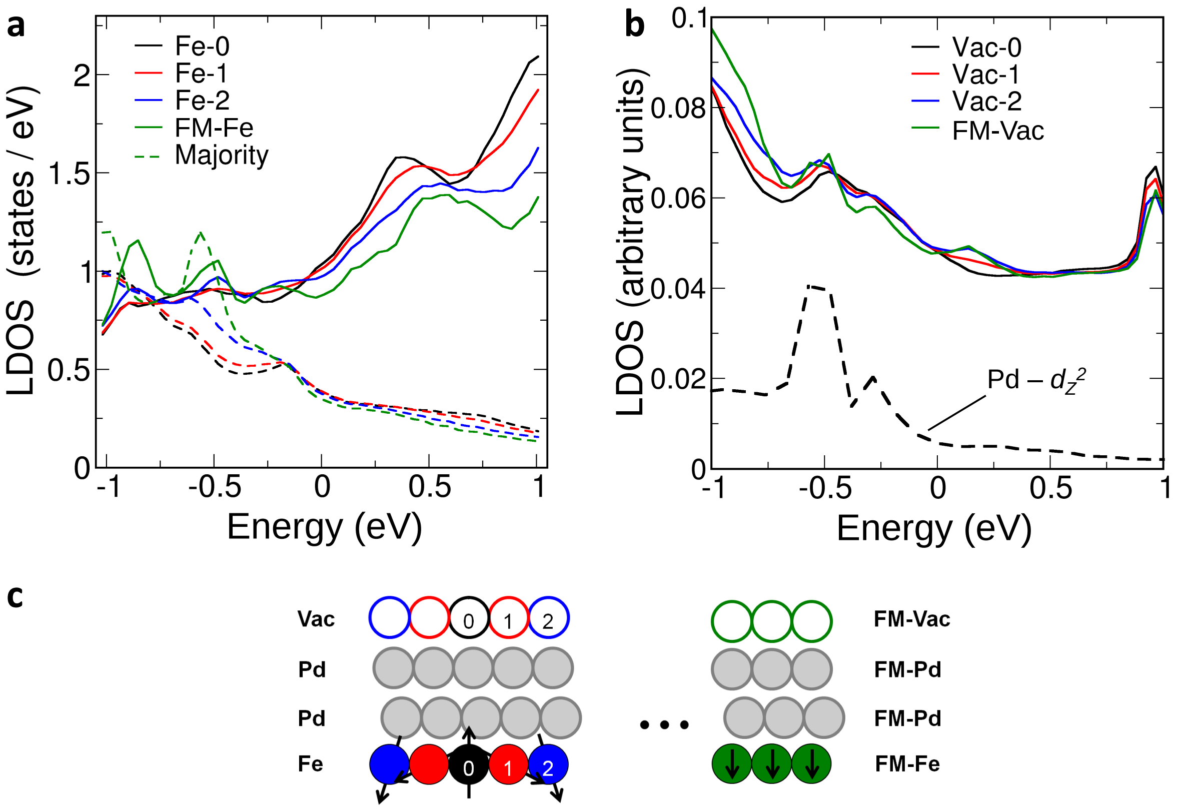

Supplementary Figure 4 | LDOS of a $D_{\mathrm{Sk}} \approx 1.7$ nm skyrmion in Pd/Pd/Fe/Ir. (a) LDOS in the magnetically-active Fe-layer resolved into minority (solid) and majority (dashed) spin-channels. The resonance-peak near $E \approx 0.5$ eV in the FM-background (green) shifts in energy when approaching the center of the skyrmion (black). **(b)** The modification of the electronic structure in Fe impacts the vacuum LDOS via hybridization through surface Pd-states. Arbitrary units are used so as to include in the same plot the nature of the Pd-d_{z^2} surface-state (black-dashed), whose features at negative energies survive in the vacuum. **(c)** Illustrative legend for (**a**, **b**) where the numbered spheres represent a line extending radially from the skyrmion's center. The vacuum domains are represented by empty spheres. FM-Fe, FM-Pd, and FM-Vac represent the unperturbed background ferromagnet.

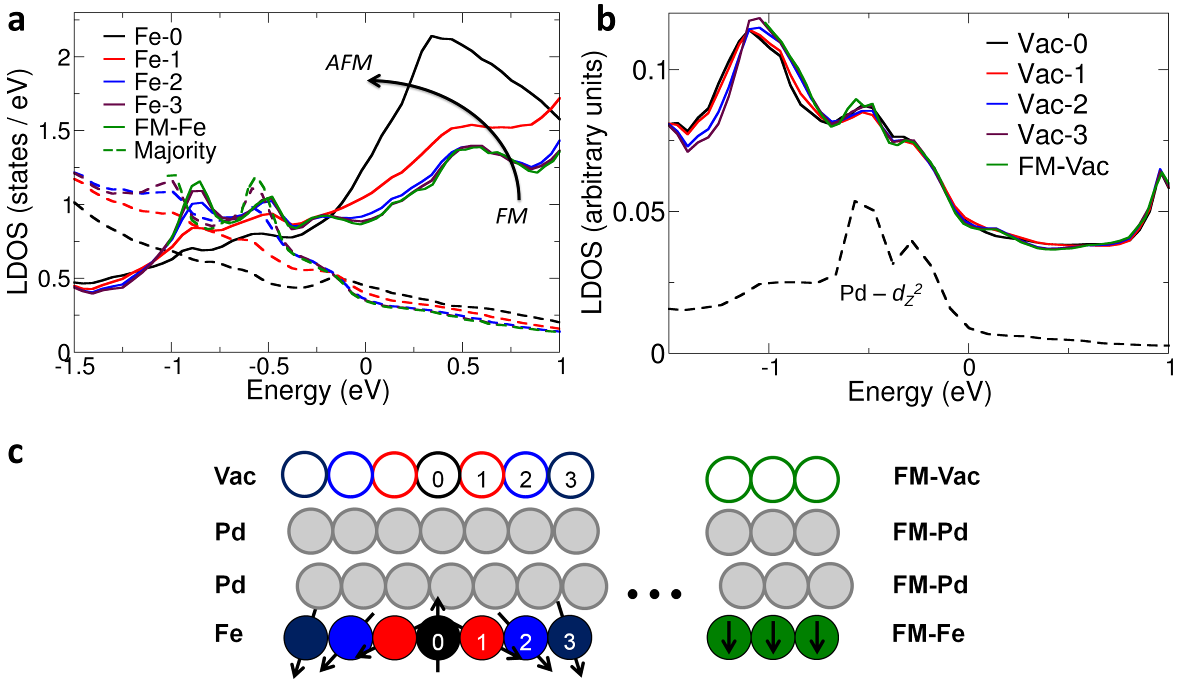

Supplementary Figure 5 | LDOS of a $D_{\mathrm{Sk}} \approx 2.2$ nm skyrmion in Pd/Pd/Fe/Ir. (a) Electronic structure in the magnetically-active Fe-layer resolved into minority (solid) and majority (dashed) spin-channels. The splitted-structure of the FM-background (green) is modified due to quasi-AFM interactions when approaching the center of the skyrmion (black). **(b)** The alteration of the LDOS in Fe impacts the vacuum structure via hybridization through surface Pd-states. Arbitrary units are used so as to include in the same plot the nature of the Pd-d_{z^2} surface-state (black-dashed). **(c)** Illustrative legend for (**a**, **b**) where the numbered spheres represent a line extending radially from the skyrmion's center. The vacuum domains are represented by empty spheres. FM-Fe, FM-Pd, and FM-Vac represent the unperturbed background ferromagnet.

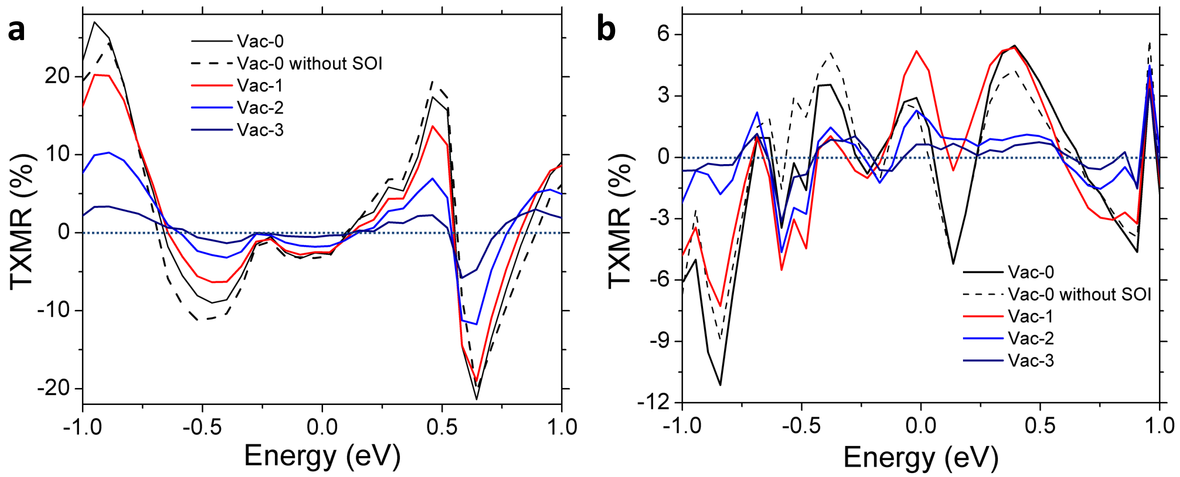

Supplementary Figure 6 | Spacially-varying TXMR in 2.2 nm skyrmions. (a) We plot the TXMR in vacuum for Pd/Fe/Ir beginning in the core of the skyrmion (Vac-0, black) and then moving radially outwards (Vac-1, red; Vac-2, blue; Vac-3, navy) towards the confining FM environment. The vacuum resonance near $0.5\ \mathrm{eV}$ (see Supplementary Figure 3b) contributes a strong peak in the TXMR near the same energy. **(b)** Again but in Pd/Pd/Fe/Ir, where the peak-signal strength appears near $-0.8\ \mathrm{eV}$, as predicted by Supplementary Figure 5b.

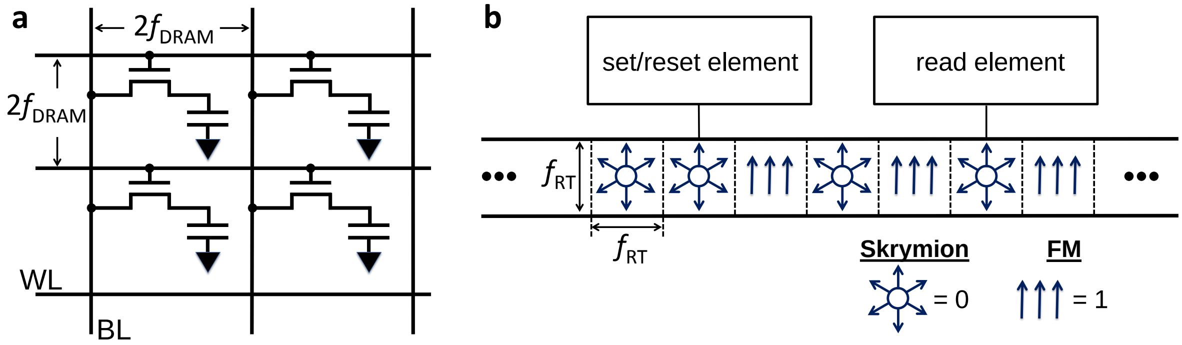

Supplementary Figure 7 | Concept spin-mixing magneto-memory versus DRAM. (a) $2{\times}2$ 1-T 1-C DRAM unit. In a random access memory, any memory cell can be accessed in roughly the same time since the WLs and BLs are connected to each bit in parallel. The ideal minimum packing footprint goes as $4f_{\mathrm{DRAM}}^2/\mathrm{bit}$ in units of area, where f_{DRAM} is the node generation. **(b)** Spin-mixing racetracks are not random access but acquire a latency associated with moving the bits sequentially out of the way to reach the requested address. However, with realistic skyrmion velocities, the access time could be roughly the same as in DRAM, while dramatically increasing the packing density.

	Z	n	Δn	$\mathrm{M_S}$	$\mathrm{M_L}$
Pd	46	45.72	−0.28	0.31	0.02
Fe	26	26.18	0.18	2.70	0.10
Ir	77	76.84	−0.16	0.02	−0.01

Supplementary Table 1 | FM state of Pd/Fe/Ir(111). The atomic number (Z), the total electronic charge (n), and the charge transfer ($\Delta n = n - Z$) are given in units of electrons. The spin-moment ($\mathrm{M_S}$) and the orbital-moment ($\mathrm{M_L}$) are given in units of μ_B.

	Z	n	Δn	$\mathrm{M_S}$	$\mathrm{M_L}$
Pd	46	45.77	−0.23	0.08	0.01
Pd	46	45.94	−0.06	0.28	0.02
Fe	26	26.20	0.20	2.63	0.09
Ir	77	76.83	−0.17	0.02	−0.01

Supplementary Table 2 | FM state of Pd/Pd/Fe/Ir(111). The atomic number (Z), the total electronic charge (n), and the charge transfer ($\Delta n = n - Z$) are given in units of electrons. The spin-moment ($\mathrm{M_S}$) and the orbital-moment ($\mathrm{M_L}$) are given in units of μ_B.

Supplementary Note 1 — Charge density, angular momentum of FM thin-films

In Supplementary Table 1, we give information regarding the charge density, charge transfer, and spin and orbital magnetic moments of the near-surface atomic layers in the ferromagnetic (FM) state in the single-Pd system (Pd/Fe/Ir).

We see that the spin magnetic moment of Fe is rather large (2.70 μ_{B}) and the orbital moment is not negligible (0.10 μ_{B}). Fe induces a sizable spin-moment in Pd of 0.31 μ_{B} which stiffens the isotropic exchange constant for first-nearest neighbors J compared to the system[1,2] having no Pd-overlayer ($J^{\mathrm{Pd/Fe/Ir}} = 14.7$ meV vs. $J^{\mathrm{Fe/Ir}} = 5.7$ meV). Pd is known to be a good Stoner system, i.e. it can easily develop a spin magnetic moment.

We also provide a similar table for the double-Pd case (Pd/Pd/Fe/Ir) in Supplementary Table 2. Interestingly in this case, the spin magnetic moments of the Fe-layer and its nearest-neighbor Pd-layer did not change greatly compared to the values obtained for Pd/Fe/Ir. However, the charge transfer of this inner Pd-layer is now much smaller. Since the surface Pd-layer interacts with Fe indirectly through the inner Pd-layer, the induced spin magnetic moment is rather small (0.08 μ_{B}) on the surface.

Supplementary Note 2 — Angular dependence of the LDOS in nano-skyrmions

We now analytically derive the change in the LDOS at site r, denoted ΔLDOS(r), inside an axially symmetric skyrmion measured from the origin at $r = 0$ as a function of energy and magnetization rotation direction defined by the unit vector of the magnetic moment $\hat{\mathbf{s}}(r) = \big(\sin\theta(r)\cos\phi(r), \sin\theta(r)\sin\phi(r), \cos\theta(r)\big)$. Examples of rotation parameters θ and dθ for given skyrmion magnetic moments can be found in Supplementary Figure 2d. When compared to the ferromagnetic state (with all moments pointing out-of-plane), there are two contributions to ΔLDOS(r), one due to spin-orbit interaction (SOI) and one due to non-collinearity (NC): $\Delta\mathrm{LDOS}(r) = \Delta\mathrm{LDOS}^{SOI}(r) + \Delta\mathrm{LDOS}^{NC}(r)$. The contribution from SOI, the so-called anisotropic magnetoresistance, is well known[3–5]:

$$\Delta\mathrm{LDOS}^{SOI}(r, E, \theta) \propto A(r, E) \cdot \left[1 - \hat{s}_z^2(r)\right] \quad , \tag{1}$$

where $A(r, E)$ is a coefficient depending on the site r and energy E, and $\hat{s}_z(r)$ is the z-component of the spin-moment at site r. Thus upon including SOI, we expect, for example, a $\sin^2\theta(r)$ dependence, which contributes to ΔLDOS(r) in second-order.

The contribution from NC, intuitively, comes from the change in the electronic structure upon rotation of the magnetic moments at consecutive sites $(i,\, j)$. For homogeneous magnetic spirals, a constant deviation in the LDOS from the ferromagnetic (FM) state will be observed for each atom in the spiral (ignoring SOI). In such a spiral, the smooth rotation of moments $\theta(i) \rightarrow \theta(j) = \theta(i) + \mathrm{d}\theta$ for each atom pair is a symmetry operation commuting with a Hamiltonian having translational invariance, making each atom equivalent, and the electronic structure the same for each atom in the spiral. However, upon transforming the spiral's pitch $\mathrm{d}\theta \rightarrow \mathrm{d}\theta'$, one would find a different constant deviation of the electronic structure from the FM-state, such that spirals of different pitch can be identified by their different magnitudes in $\Delta\mathrm{LDOS}^{NC}$.

In skyrmions, however, the rotation of the magnetic moments is not homogeneous, i.e. $\mathrm{d}\theta$ is not constant for all nearest-neighbor atom pairs while traversing the skyrmion's diameter. Thus there will be a site-dependent deviation in the LDOS among the atoms inside a skyrmion with respect to each other. In what follows, we demonstrate that these deviations are a complex function of the rotation angles, which will depend on the details of the electronic structure, the energy probed, and even the size of the skyrmions.

To derive $\Delta\mathrm{LDOS}^{NC}(r)$, we utilize multiple scattering theory: G is the Green function describing the whole system upon rotation of the magnetic moments, and g is the Green function describing the initial FM-state. The Green function will be used to evaluate the change in the LDOS induced by the rotation of the magnetic moments:

$$\Delta\mathrm{LDOS}^{NC}(r, E) = -\frac{1}{\pi}\, \Im\left\{\mathrm{Tr}_{\mathrm{LS}}\left[\Delta G^{rr}(E)\right]\right\} \quad , \tag{2}$$

as given in a matrix notation where a trace over orbital and spin angular momenta has to be performed. G can be evaluated via the Dyson equation connecting the non-collinear state to the ferromagnetic one:

$$G = g + g\Delta V G = g + g\Delta V g + g\Delta V g\Delta V g + ... \tag{3}$$

where ΔV describes the change of the potential upon rotation of the magnetic moments. It can be expressed as:

$$\Delta V(r) = V_{\mathrm{diff}}(r)\, (\boldsymbol{\sigma} \cdot \hat{\mathrm{s}}(r) - \sigma_z) \quad , \tag{4}$$

where $\boldsymbol{\sigma}$ is the vector of Pauli matrices, and V_{diff} is the difference of the two spin components of the ferromagnetic potential $(V_0^{\uparrow} - V_0^{\downarrow})/2$.

We execute a similar expansion for the ferromagnetic initial Green function matrix g:

$$g = g_{\mathrm{sum}} 1_2 + g_{\mathrm{diff}} \sigma_z \quad , \tag{5}$$

where 1_2 is the 2×2 identity matrix.

Let us evaluate the first-order and second-order terms contributing to the Dyson equation (Supplementary Equation 3):

$$\Delta G^{rr} = \sum_i g^{ri} \Delta V^i g^{ir} + \sum_{ij} g^{ri} \Delta V^i g^{ij} \Delta V^j g^{jr} + ... \tag{6}$$

where i and j are sites surrounding site r, or can be the site r itself. Since the trace over spin has to be performed, we will focus only on the terms that in the end will contribute to Supplementary Equation 2. We use a pair of useful properties of the Pauli matrices:

$$\mathrm{Tr_S}\big[\sigma_x\big] = \mathrm{Tr_S}\big[\sigma_y\big] = \mathrm{Tr_S}\big[\sigma_z\big] = 0 \ \ , \tag{7}$$

and

$$(\boldsymbol{\sigma} \cdot \hat{\mathrm{s}})(\boldsymbol{\sigma} \cdot \hat{\mathrm{s}}') = \hat{\mathrm{s}} \cdot \hat{\mathrm{s}}' + \mathrm{i}\ \boldsymbol{\sigma} \ \cdot (\hat{\mathrm{s}} \times \hat{\mathrm{s}}') \ \ , \tag{8}$$

where i is the imaginary unit. After simplifying, we find the following result:

$$-\frac{1}{\pi}\Im\Big\{\mathrm{Tr_{LS}}\big[g^{ri}\Delta V^i g^{ir}\big]\Big\} = B^{rir}(E)\,[1 - \hat{s}_z(i)] \tag{9}$$

where the coefficient B is given by

$$B^{rir} = \frac{2}{\pi}\Im\Big\{\mathrm{Tr_L}\big[g^{ri}_{\mathrm{sum}} V^i_{\mathrm{diff}} g^{ir}_{\mathrm{diff}} + g^{ri}_{\mathrm{diff}} V^i_{\mathrm{diff}} g^{ir}_{\mathrm{sum}}\big]\Big\} \ \ . \tag{10}$$

In other words, the first sum in Supplementary Equation 6 leads to a behavior like $(1-\cos\theta_i)$.

The second-order term is given by

$$-\frac{1}{\pi}\Im\Big\{\mathrm{Tr_{LS}}\big[g^{ri}\Delta V^i g^{ij}\Delta V^j g^{jr}\big]\Big\} = C^{rijr}\big[\hat{\mathbf{s}}(i)\cdot\hat{\mathbf{s}}(j) - \big(\hat{s}_z(i) + \hat{s}_z(j)\big) + 1\big] \ \ , \tag{11}$$

where the coefficient C is related to the Green functions and V_{diff} by:

$$\begin{aligned} C^{rijr} = -\frac{2}{\pi}\Im\Big\{\mathrm{Tr_L}\big[& g^{ri}_{\mathrm{sum}} V^i_{\mathrm{diff}} g^{ij}_{\mathrm{sum}} V^j_{\mathrm{diff}} g^{jr}_{\mathrm{sum}} \ + \ g^{ri}_{\mathrm{diff}} V^i_{\mathrm{diff}} g^{ij}_{\mathrm{diff}} V^j_{\mathrm{diff}} g^{jr}_{\mathrm{sum}} \\ & + g^{ri}_{\mathrm{sum}} V^i_{\mathrm{diff}} g^{ij}_{\mathrm{diff}} V^j_{\mathrm{diff}} g^{jr}_{\mathrm{diff}} \ + \ g^{ri}_{\mathrm{diff}} V^i_{\mathrm{diff}} g^{ij}_{\mathrm{sum}} V^j_{\mathrm{diff}} g^{jr}_{\mathrm{diff}}\big]\Big\} \ \ . \end{aligned} \tag{12}$$

Thus we obtain a dependence on the dot product of the unit vectors of the magnetic moments $(1 - \cos \mathrm{d}\theta \cos \mathrm{d}\phi)$ and a contribution depending only on the z-components of the unit vectors of the magnetic moments.

We have thus demonstrated that due to NC, the dependence of the change in the LDOS with respect to the ferromagnetic state upon rotation of the magnetic moments is not trivial, and will have terms depending on the dot product between magnetic moments, contrary to the contribution coming from SOI. The non-collinear contribution is then

$$\Delta\mathrm{LDOS}^{NC}(r, E, \{\mathbf{s}\}) = \sum_i B^{rir}(E)\big(1 - \hat{s}_z(i)\big) + \sum_{ij} C^{rijr}(E)\big[\hat{\mathbf{s}}(i)\cdot\hat{\mathbf{s}}(j) - \big(\hat{s}_z(i) + \hat{s}_z(j)\big) + 1\big] \ , \tag{13}$$

where $\{\mathbf{s}\}$ is the spin configuration. Of course, depending on the details of the electronic structure and strength of perturbation related to the non-collinearity, higher-order terms can be important and have to be included in Supplementary Equation 13.

Combining $\Delta\mathrm{LDOS}^{NC}$ and $\Delta\mathrm{LDOS}^{SOI}$, in the next section we will fit the change in the LDOS in terms of trigonometrical functions that depend on the rotation angles of the magnetic moments. We will apply these fits to our *ab initio* results as well as to an extended Alexander-Anderson model used to interpret the variation of the LDOS resonance-splitting upon rotation of the magnetic moments on neighboring sites.

Supplementary Note 3 — Two-atom extended Alexander-Anderson model

We wish to estimate the change in the LDOS and qualitatively understand the shifting in energy of resonant d-states in Fe as a function of the rotation angle between adjacent moments. To this end, we consider for simplification two magnetic atoms $(i,\ j) = (1,\ 2)$ each having one localized orbital d_{z^2} whose single-particle eigenenergy is centered about $E = \epsilon$. The initial Hamiltonian describing this model is diagonal in spin-space. We could also consider an orbital of the type d_{xz} in order to address the coupling induced by SOI between the d_{z^2} and d_{xz}, as done by Caffrey *et al*[6]. However, since the impact of SOI on the LDOS has already been discussed by others, we focus here on the impact of NC on the LDOS. We study the ΔLDOS as we vary $\mathrm{d}\theta = \theta_1 - \theta_2$ between the two atoms. We restrict the hopping from atom-to-atom to non-spin-flip processes, characterized by the interaction parameter V_{hop}.

In terms of Green functions, the following equation gives the LDOS for site 1:

$$\mathrm{LDOS}(1; E, \{\mathbf{s}\}) = -\frac{1}{\pi}\Im\Big\{\mathrm{Tr}_{\mathrm{S}}\big[G_{11}(E)\big]\Big\} = -\frac{1}{\pi}\Im\Big\{\mathrm{Tr}_{\mathrm{S}}\left[E - H + i\Gamma\right]^{-1}\Big\}_{11} \ , \tag{14}$$

where Γ takes care of the broadening of the states. Instead of solving exactly the previous equation, one could also use perturbation theory, as described in the previous Supplementary Note 2, simplifying Supplementary Equation 13 to:

$$\Delta\mathrm{LDOS}^{NC}(1;E,\{\mathbf{s}\}) = D(E)\cdot(1-\cos\mathrm{d}\theta)\,, \tag{15}$$

where $D = B^{121} + C^{1221}$.

The energy of the resonant d-states, their width, and splittings come from our first-principles calculations, e.g. Supplementary Figure 3a (atom Fe-3, navy curve). To obtain the proper splitting we choose a hopping parameter $V_{\mathrm{hop}} \approx$ 300-400 meV. We show the resulting LDOS in Supplementary Figure 1 for five different rotation angles $\mathrm{d}\theta$. There we reproduce the splitting of the resonance-peaks that we have seen in our first-principles calculations, where *d-d* hybridization is important, as seen in e.g. Figure 3a of the main text, or Supplementary Figures 3a, 4a, and 5a of the Supplementary Information.

For the limiting cases of a ferromagnetic state and an antiferromagnetic state, we recover the expectations of the Alexander-Anderson model[7,8]. Indeed, when the two atoms are in the ferromagnetic state ($\mathrm{d}\theta = 0°$), the *d-d* hybridization leads to a splitting of the original single orbital into bonding- and antibonding-states, seen as broad resonances near 0.5 eV and 1.25 eV, respectively (Supplementary Figure 1, green curve). The splitting is then given by $2V_{\mathrm{hop}}$.

If the magnetic state is antiferromagnetic ($\mathrm{d}\theta = 180°$), there is repulsion between the minority and majority spin-states leading to a shift given by $V_{\mathrm{hop}}/(E^{\downarrow}_{i,dz^2} - E^{\uparrow}_{i,dz^2})$. In our simple model, $E^{\downarrow}_{i,dz^2} - E^{\uparrow}_{i,dz^2}$ is extremely large, thus the shift is not observed in Supplementary Figure 1. It is interesting to see how the splitting between the resonance-peaks decreases upon rotation of the magnetic moments until they merge to a single resonance in the antiferromagnetic case (Supplementary Figure 1, black curve). This is in accordance with the behavior of the LDOS calculated from first-principles for different sized skyrmions in the two systems Pd/Fe/Ir (main manuscript Figure 3a and Supplementary Figure 3a) and Pd/Pd/Fe/Ir (Supplementary Figures 4a and 5a).

Next, we wish to estimate the change in the LDOS as a function of rotation as previously discussed, which leads to the TXMR signal. In Supplementary Figure 2 we plot the change in LDOS compared against the background-FM for the model along with our *ab initio* results in Pd/Pd/Fe/Ir for skyrmions 1.7 nm and 2.2 nm in diameter, respectively. In addition we

show fitted functions against θ and dθ. Interestingly, a good fit to the change in LDOS shown in Supplementary Figure 2a,b is found by considering

$$\Delta\text{LDOS}(r; E, \{\mathbf{s}\}) \approx A(E) \cdot \sin^2\theta(r) + D(E) \cdot \big[1 - \cos\theta(r)\big] \tag{16}$$

instead of strictly employing the terms given by Supplementary Equation 13. This result is similar to what we found in the simple two-orbital Alexander-Anderson model (Supplementary Equation 15). In such systems, the contribution from all spin-moment dot products (see Supplementary Equation 11) behaves on average like $\cos(\theta)$. This is naturally satisfied in the two-orbital model.

In Supplementary Figure 2a the change in LDOS at the Fermi energy $E = E_\mathrm{F} = 0$ is depicted. The blue dots represent the total ΔLDOS given from our *ab initio* calculations for each atom extending radially from the skyrmion core. The fitting-function $f(\theta)$ is Supplementary Equation 16 and shown to be effective in fitting the first-principles data. We note that the change in the LDOS at the Fermi energy was not large. This is different than the situations in Supplementary Figure 2b,c where we probed the energy resonances near 0.5 eV, and a larger change in the LDOS was induced. In Supplementary Figure 2b, the fitting function $f(\theta)$ is again Supplementary Equation 16 and shown to be effective in fitting the *ab initio* data.

In Supplementary Figure 2c, we plot the ΔLDOS as given by the two-atom Alexander-Anderson model (black curve) with two fitting-functions. The first-order fit (red-dashed curve) based on Supplementary Equation 15 is shown to be slightly inaccurate when fitting the model ΔLDOS. Instead a higher-order term is needed to fit the data (blue-dashed curve). Thus we learned from the Alexander-Anderson model that depending on the probed energy, additional terms can be needed to improve the fit. A term proportional to $\sin^2(\theta)$, similar to the one expected when SOI is included, improves considerably the fit to the non-collinear contribution to ΔLDOS. For example, near energy resonances, especially if they are sharp, the perturbative expansion up to second-order from Supplementary Note 2 begins to break down and higher-order terms are necessary to improve the fitting-functions.

Supplementary Note 4 — LDOS in single-Pd 2.2 nm skyrmions

Let us now turn to the case of a single-Pd system but for a larger skyrmion, $D_{\mathrm{Sk}} \approx$ 2.2 nm, compared to the one shown in the main manuscript ($D_{\mathrm{Sk}} \approx 1.7$ nm, Figure 3). In Supplementary Figure 3, we plot the LDOS in a realistic nano-skyrmion in Pd/Fe/Ir(111) for the magnetically-active Fe-layer (**a**) and its corresponding vacuum sites (**b**), but with a slightly larger energy window $[-1.5, +1.5]$ eV as compared to the main text, where a window of $[-1.0, +1.0]$ eV was analyzed.

Similar to the case of the smaller skyrmion in the single-Pd system as shown in the main manuscript (Figure 3a, FM-Fe, green curve), a large resonance peak is observed in the background-FM Fe-film at 0.5 eV (Supplementary Figure 3a, FM-Fe, green curve). But by increasing the energy window beyond 1.0 eV, we clearly see a second resonance appearing at 1.25 eV in the nearly-FM atom Fe-3 at the edge of the skyrmion (Supplementary Figure 3a, Fe-3, navy curve). The appearance of these two resonance peaks in tandem suggests a strong interaction due to ferromagnetic coupling. In other words, the resonant-peaks at 0.5 eV and 1.25 eV are actually the result of FM-interactions which have split a single resonance in two. An analogy can be made between these FM-splitted resonance-peaks and localized degenerate atomic orbitals at different sites which upon interaction split in energy between bonding- and antibonding-states. The essential physics of this process were reproduced within an extended Alexander-Anderson model for *d*-*d* hybridization between FM-Fe *d*-states[7,8] in the previous Supplementary Note 3.

The splitting in energy between the two resonance-peaks reduces when the rotation angle of the magnetic moment increases (Supplementary Figure 3a, Fe-2, blue curve, and Fe-1, red curve). Finally, in the central spin-flipped atom of the skyrmion (Supplementary Figure 3a, Fe-0, black curve), the splitting almost disappears as the two resonance-peaks quasi-merge. This is evidence of antiferromagnetic coupling between the central atom and its nearest-neighbors[7,8]. This coupling was also reproduced within our Alexander-Anderson model in the antiferromagnetic case in the previous Supplementary Note 3.

An important difference with respect to the center of the skyrmion (Supplementary Figure 3a, Fe-0, black curve) is observed when comparing to the smaller $D_{\mathrm{Sk}} \approx 1.7$ nm case (main muscript Figure 3a, Fe-0, black curve). In the smaller skyrmion, the quasi-merged resonance still shows two separate peaks, while in the larger skyrmion there is only a sin-

gle, broad resonance peak. Thus we expect a difference in the TXMR signal in the larger skyrmion compared to the $D_{\mathrm{Sk}} \approx 1.7$ nm case. In general, there will be a weak size dependence of the spin-mixing signal, which should slowly decay as the diameter of the skyrmion is increased. In addition, from Supplementary Figure 3b we can see that the vacuum resonance near 0.5 eV in the smaller defect (main manuscript Figure 3b, solid curves) survives in the larger skyrmion (Supplementary Figure 3b, solid curves), and the TXMR remains detectible, as seen in Supplementary Figure 6a.

To complete our analysis of the LDOS shown in Supplementary Figure 3, we mention that with regards to magnifying the strength of the TXMR effect, we need not restrict ourselves only to the energy window near 0.5 eV, but also look for other energy windows where a large change in the LDOS is observed as a function of position. We see this in the majority-states near -1.0 eV (Supplementary Figure 3a, dashed-curves), where the spin-mixing effect is perhaps even stronger than in the 0.5 eV window composed of minority-states. Indeed, the TXMR signal is shown to be large for negative bias energies near -1.0 eV in this system (see main manuscript Figure 4a,b and Supplementary Figure 6a).

Supplementary Note 5 — LDOS of skyrmions in the double-Pd systems

In Supplementary Figure 4, we plot the LDOS in a skyrmion in Pd/Pd/Fe/Ir(111) with diameter $D_{\mathrm{Sk}} \approx 1.7$ nm for the magnetically-active Fe-layer (**a**) and its corresponding vacuum sites (**b**).

Similar to the case of the smaller skyrmion in the single-Pd system as shown in the main manuscript (Figure 3a, FM-Fe, green curve), a strong resonance-peak appears near 0.5 eV composed of minority-spin channel d-states (Supplementary Figure 4a, FM-Fe, green curve). This resonance-peak shifts in energy upon increased rotation of the magnetic moments moving towards the center of the skyrmion (Supplementary Figure 4a, Fe-2, blue curve; Fe-1, red curve; and finally Fe-0, black curve). This observed change in the electronic structure as a function of position due to the spin-mixing of majority- and minority-states suggests there will be a TXMR signal in this system as shown previously for the single-Pd system.

Contrary to the single-Pd case, however, the resonance in the hybrid Fe-Pd-spd_{z^2} state (Pd-d_{z^2} for short) observed in the Pd-overlayer around 0.5 eV vanishes, and instead appears near -0.5 eV (Supplementary Figure 4b, black-dashed curve). The steepness of this reso-

nance leads to a disturbance in the vacuum-LDOS as a function of position in slightly more negative energies, near -0.8 eV (Supplementary Figure 4b, solid curves). The character of the hybrid Pd-d_{z^2} surface-state leads to a flat region in the vacuum-LDOS at positive energies. We then expect here a smaller TXMR signal in comparison to the single Pd-system for positive bias voltages. Therefore, in the double-Pd system, we suggest probing negative bias energies near -0.8 eV, where the spin-mixing effect is more significant, as exemplified in main manuscript Figure 4d,e.

In Supplementary Figure 5, we plot the LDOS for a larger skyrmion, $D_{\mathrm{Sk}} \approx 2.2$ nm, in Pd/Pd/Fe/Ir(111). We use a similar labeling convention when decomposing the LDOS. Interestingly, the d-resonances are broader (Supplementary Figure 5a, solid curves) than for the smaller skyrmion (Supplementary Figure 4a, solid curves). In the center of the skyrmion, only one broad d-resonance is observed (Supplementary Figure 5a, Fe-0, black curve). To reiterate, this indicates that the TXMR signal will be different upon increasing the diameter of any skyrmion since its electronic structure is modified upon increasing D_{Sk}. From the shape of the vacuum-LDOS (Supplementary Figure 5b, solid curves), the TXMR is nicely detectable at an energy range around -0.8 eV, as confirmed in Supplementary Figure 6b, and in the same energy window as the smaller $D_{\mathrm{Sk}} \approx 1.7$ nm Pd/Pd/Fe/Ir(111) case.

From Supplementary Notes 4 and 5 we can conclude that for each skyrmion in a Pd/Fe/Ir(111) or Pd/Pd/Fe/Ir(111) system, we can select an energy region in which a large tunneling spin-mixing magnetoresistance (TXMR) will be found. Due to the relevant energy of detection, one can distinguish skyrmions of different sizes, and discriminate between skyrmions in Pd-films of different thicknesses. Finally, and perhaps most importantly, the spatial-variation of the spin-mixing signal means that the internal atomic structure of each individual skyrmion can be resolved. This means the TXMR can be used to visualize the size, shape, and structure of individaul defects. In devices based on spin-mixing, this also means that skyrmions can be used as nano-scopic information carriers, where the TXMR would be used to read the magnetic state-of-bit.

Supplementary Note 6 — TXMR in larger 2.2 nm skyrmions

In Supplementary Figure 6 we plot the TXMR signal spatially-resolved for the 2.2 nm skyrmions in both systems. The spin-mixing signals retain the same general features and

shapes as in the smaller defects (main manuscript Figure 4a-d). The important peak near 0.5 eV in the Pd/Fe/Ir case remains robust (Supplementary Figure 6a), along with another strong peak near −1.0 eV. In the case of Pd/Pd/Fe/Ir (Supplementary Figure 6b), the TXMR has the strongest signal near −0.8 eV, as before. Therefore one could infer from Supplementary Figure 6 that as the diameter of skyrmionic quasiparticles is increased, the spin-mixing effect not only survives, but also that specific locations of strong TXMR signals remain in similar energy windows as in smaller structures.

Supplementary Note 7 — Skyrmion racetracks for dense magnetic memories

Spin-transfer torque magnetic random access memory (STT-MRAM) circuits reliably read-out bit-states depending on a tunneling magnetoresistance anisotropy of ∼30-50% in some structures[9], with a hope to achieve a magnetoresistance ratio $R_{\mathrm{ON}}/R_{\mathrm{OFF}} \approx 200\%$ by 2022[10]. A TXMR effect as large as ∼20% as we have shown in this work should be enough to provide adequate read-margin for scaled technologies, and is larger than the $< 2\%$ change in resistance found in widespread commercially-used hard disk read heads based on anisotropic magnetoresistance alone[11]. Smaller changes in magnetoresistance just means there should be a more sensitive read-out circuit. Typically this means a few extra control- and boost-transistors and does not substantially increase the footprint of the memory, i.e. incorporating more sensitive read hardware does not degrade packing density considerably.

In potential skyrmion-based devices using CPP-TXMR, a $R_{\mathrm{ON}}/R_{\mathrm{OFF}} \approx 120\%$ could feasably be well-worth the tradeoff when considering the possible performance gains with regards to: (1) potentially very low power dissipation due to small currents needed to manipulate the magnetic textures; (2) fast speed operation due to reduced read/write latencies associated with nano-scopic size; and (3) large increases in packing density. Let's consider points (2) and (3) in greater detail.

One issue with racetrack memories is that they are not random access. In a random access memory (RAM), any read/write operation can access any bit with roughly the same access time since the word and bit access lines (WL and BL), which are connected to the set/reset elements and read elements, are also connected in parallel with the individual memory cells (see Supplementary Figure 7a). In a racetrack memory, the situation is different. In practice, if a read/write were requested for an address whose representative bit were at the

end of the racetrack, there would be additional latency associated with moving subsequent magnetic domains out the way until the requested bit were under the read or write device (see Supplementary Figure 7b). But does this make racetrack memory intrinsically slower than RAM?

This is not clear. It depends on the velocities of the skyrmions in the racetrack. Consider 200 nm length racetracks populated with 2 nm in diameter quasiparticles in an array of 100 domains (or 100 bits). With velocity[12] $v_{\mathrm{Sk}} = 100$ m/s , the furthest bit would acquire an additional delay in time t_{delay}

$$t_{\mathrm{delay}} = \frac{L_{\mathrm{RT}}}{v_{\mathrm{Sk}}} \approx \frac{\left(2\,\frac{\mathrm{nm}}{\mathrm{Sk}}\right)\left(1\,\frac{\mathrm{Sk}}{\mathrm{bit}}\right)(100\ \mathrm{bits})}{100\left(\frac{\mathrm{m}}{\mathrm{s}}\right)} = 2\ \mathrm{ns}\quad , \tag{17}$$

a small number compared to the total access time needed to complete the read or write operation, which in modern dynamic RAM (DRAM) is in the range 20–50 ns[13]. We do note, however, that larger in-plane currents will be required to accelerate the quasiparticles up to a velocity such as $v_{\mathrm{Sk}} = 100$ m/s, meaning there will be a tradeoff between t_{delay} and power consumption.

Regardless, by incorporating skyrmion racetracks based on spin-mixing, there seems to be at first glance negligible additional acquired access latency – in fact, we may learn to find in the end that racetracks can be potentially faster than traditional RAM in certain geometries and biasing conditions, due to the nano-scopic size of the skyrmion quasiparticles.

With regards to circuit layouts, it is clear that moving to racetracks will provide large gains in packing density. As an example, let's compare the workhouse 1-transistor 1-capacitor (1-T 1-C) DRAM unit to our racetrack-based spin-mixing magneto-memory. Considering the lithographical node generation, or minimum feature size, f, DRAM memory minimum packing requirements for a single bit's footprint in the ideal case goes as surface area $\mathrm{SA}_{\mathrm{DRAM}} \approx 4f^2$ per bit (Supplementary Figure 7a). The current technology node for DRAM in 2015 is given as $f_{\mathrm{DRAM}} \approx 22$ nm by the International Technology Roadmap for Semiconductors (ITRS)[10]. In a skyrmion racetrack, the potential effective per-bit surface area could be reduced to possibly $\mathrm{SA}_{\mathrm{RT}} \approx f_{\mathrm{RT}}^2$ per bit, where f_{RT} is the diameter of the magnetic skyrmions in the racetrack (Supplementary Figure 7b). In our study thus far we considered realistic skyrmions of order $D_{\mathrm{Sk}} \approx 2$ nm. Comparing against current DRAM

arrays, we define the potential gain in packing density Γ as

$$\Gamma \approx \frac{SA_{\mathrm{DRAM}}}{SA_{\mathrm{RT}}} \approx \frac{4f_{\mathrm{DRAM}}^2}{f_{\mathrm{RT}}^2} = \frac{(4)(22\ \mathrm{nm})^2}{(2\ \mathrm{nm})^2} = 500\ . \tag{18}$$

Even including more intricate sense amplifiers for read/write operations in skyrmion racetracks, it is clear that incorporation of sub-5 nm skyrmions as data-carriers would substantially increase packing density compared to current state-of-the-art technologies.

Possibly the gains in packing density could even be larger introducing vertical racetracks[14], which are difficult to fabricate thus far.

Supplementary References

1. Dupé, B., Hoffmann, M., Paillard, C. & Heinze, S. Tailoring magnetic skyrmions in ultra-thin transition metal films. *Nature Commun.* **5**, 4030 (2014).

2. Heinze, S. *et al.* Spontaneous atomic-scale magnetic skyrmion lattice in two dimensions. *Nature Phys.* **7**, 713-718 (2011).

3. Bode, M. *et al.* Magnetization-direction-dependent local electronic structure probed by scanning tunneling spectroscopy. *Phys. Rev. Lett.* **89**, 237205 (2002).

4. Gould, C. *et al.* Tunneling anisotropic magnetoresistance: a spin-valve-like tunnel magnetoresistance using a single magnetic layer. *Phys. Rev. Lett.* **93**, 117203 (2004).

5. von Bergmann, K. *et al.* Tunneling anisotropic magnetoresistance on the atomic scale. *Phys. Rev. B* **86**, 134422 (2012).

6. Caffrey, N. M. *et al.* Tunneling anisotropic magnetoresistance effect of single adatoms on a noncollinear magnetic surface. *J. Phys.: Condens. Matter* **26**, 394010 (2014).

7. Alexander, S. & Anderson, P. W. Interaction Between Localized States in Metals. *Phys. Rev. A* **133**, A1594-A1603 (1964).

8. Oswald, A., Zeller, R., Braspenning, P.J. & Dederichs, P. H. Interaction of magnetic impurities in Cu and Ag. *J. Phys. F. Met. Phys.* **15**, 193-212 (1985).

9. Tehrani, S. T. *et al.* Magnetoresistive random access memory using magnetic tunnel junctions. *Proceedings of the IEEE* **91**, 703-714 (2003).

10. *The International Technology Roadmap for Semiconductors*, http://www.itrs.net (2013).

11. Pohm, A. V., Huang, J. S. T., Daughton, J. M., Krahn, D. R. & Mehra, V. The design of a one megabit nonvolatile M-R memory chip using 1.5x5 μm cells. *IEEE Trans. Magn.* **24**, 3117-3119 (1988).

12. Fert, A., Cros, V. & Sampaio, J. Skyrmions on the track. *Nature Nanotech.* **8**, 152-156 (2013).

13. Son, Y. H., Seongil, O., Ro, Y., Lee, J. W. & Ahn, J. H. Reducing memory access latency with asymmetric DRAM bank organizations. *Proceedings of the 40th Annual International Symposium on Computer Architecture*, 380-391. June 23-27, Tel-Aviv, Isreal (2013).

14. Parkin, S. S. P., Hayashi, M. & Thomas, L. Magnetic domain-wall racetrack memory. *Science* **320**, 190-194 (2008).
